# Characterization of Biofilm Formed by Phenanthrene-Degrading Bacteria on Rice Root Surfaces for Reduction of PAH Contamination in Rice

**DOI:** 10.3390/ijerph16112002

**Published:** 2019-06-05

**Authors:** Yuman Zhou, Xiaorong Gao

**Affiliations:** Liaoning Key Laboratory of Molecular Recognition and Imaging, School of Bioengineering, Dalian University of Technology, Dalian 116023, China; 17824826805@163.com

**Keywords:** biofilm, root surfaces, hydrophobicity, *Pseudomonas* sp. JM2-gfp, phenanthrene, degradation

## Abstract

One effective method in to reduce the uptake of organic contaminants by plants is the development of a root barrier. In this study, the characterization of biofilm structure and function by phenanthrene-degrading *Pseudomonas* sp. JM2-gfp on rice root surfaces were carried out. Our results showed that root surfaces from three rice species, namely *Liaojing*401, *Koshihikari*, and *Zhenzhuhong* all present hydrophobicity and a high initial adhesion of strain JM2-gfp. Matured robust biofilm formation occurred at 48 h on the root surfaces. The biofilm exhibited cell dense aggregates and biomass embedded in the extracellular polymeric substance (EPS) matrix. EPS composition results showed that the proteins, carbohydrates, lipids and nucleic acids are produced in the biofilm, while the content varied with rice species. Under the initial concentration of phenanthrene 50 mg·L^−1^, the residual phenanthrene in plant roots from ‘*Zhengzhuhong*’, ‘*Koshihikari*’ and ‘*Liaojing*401’ with biofilm mediated were significantly decreased by 71.9%, 69.3% and 58.7%, respectively, compared to those without biofilm groups after 10 days of exposure. Thus, the biofilm colonized on roots plays an important role of degradation in order to reduce the level of phenanthrene uptake of plants. Thereby, the present work provides significant new insights into lowering the environmental risks of polycyclic aromatic hydrocarbons (PAHs) in crop products from contaminated agriculture soils.

## 1. Introduction

With polycyclic aromatic hydrocarbons (PAHs) in agriculture, soil contamination is of great worldwide concern because of their persistent organic lethal nature [1]. Crops grown in PAH-contaminated soil uptake PAHs (phenanthrene and pyrene) that accumulate in the edible parts, posing a substantial threat to human health. Bacterial biodegradation is regarded as a more effective approach compared to the chemical and physical methods in order to address PAH-contaminated sites for the removal of PAHs [2]. Numerous strains of microorganisms with the capability to degrade PAHs have been successfully isolated [3]. However, the low bioavailability caused by their poor survival rate in soil has greatly limited the application of degrading-bacteria in the bioremediation of PAHs 

Recent studies have highlighted that certain PAH-degrading microorganisms are able to form a biofilm on the surfaces of plant roots, leading to the accumulation of high biomass and its long-term survival for continuous degradation in rhizosphere soil, thus reducing the absorption of PAHs by roots and ensuring the safety of crop products [4,5]. However, to our knowledge, in situ PAH-degrading biofilm on the root surfaces has not been observed.

Previous extensive work has been performed on biofilm formation using solid surfaces. Accumulated experimental evidence has shown that the hydrophobicity, roughness and the charges of interacting surfaces are essential for processes such as attachment, self-assembly and dispersion during the formation of biofilm [6,7]. For example, microbial adhesion strongly depends on the hydrophobic–hydrophilic structures of the interacting surfaces, increasing cell surface and the hydrophobicity of interacting surfaces, which facilitates cell immobilization and triggers the specific forces responsible for the irreversible adhesion [8]. However, there are different views on the impacts of the hydrophobicity of bacteria and supporting surfaces on biofilm formation [8,9]. It has been discovered that micro-organisms are more likely to adhere to hydrophobic materials than hydrophilic ones [10,11,12,13]. De Oliveira observed that *Salmonella* adhered more easily to hydrophobic materials (such as polystyrene) than to glass. Glass is the least favorable material for the development of biofilm [14]. In contrast, in another study conducted by Rodriguez-Melcon adhesion behaved differently, showing higher numbers of biofilm formation on hydrophilic materials (e.g. glass) than on hydrophobic surfaces (e.g. polystyrene) [15,16]. Conditions and parameters need to be investigated and optimized for the stable and sustainable functioning of biofilm. So far, relatively few studies have addressed the formation of biofilm on living substrates [17]. 

A root surface, namely epidermis, is the interface between the plant roots and the external soil environment contains living microorganisms. Root epidermis serves several functions, such as the continuous secretion of metabolic compounds and absorption of water and mineral nutrients. These characteristics are beneficial to both the uptake of contaminates in plants from the rhizosphere and the biofilm formation of the microorganism [18,19]. For example, plant polysaccharides stimulate the formation of *Bacillus subtilis* biofilm [19]. Maize root exudates promote the formation of biofilm of *Bacillus amyloides* SQR9 by promoting cell growth and inducing the production of extracellular matrix [20]. However, it is not clear whether the hydrophobicity of root surfaces affect the biofilm formation [21,22]. 

Our study aims to investigate the effects of rice root surface characteristics on bacterial biofilm formation. By introducing phenanthrene-degrading bacteria JM2-gfp, a functional interface for the prevention of organic pollutant uptake in crops was formed, and the safety of agricultural products was thereby ensured.

## 2. Materials and Methods

### 2.1. Materials

Glass and polystyrene were cut into 1 cm ×1 cm pieces, sterilized with 70% ethanol for 15 min, and then rinsed with sterile water before use. Stock strain JM2-gfp was from phenanthrene-degrading bacterium *Pseudomonas* sp. JM2, labeled with GFP as visual marker [23].

Analytical grade phenanthrene (≥97%) was purchased from Tokyo Chemical Industry Co., Ltd. (Shanghai, China). Methyl alcohol, n-hexane and acetone of HPLC grade were purchased from J&K Scientific Ltd. (Beijing, China).

### 2.2. Plants Preparation

The cultivated rice seeds of ‘*Liaojing*401’, ‘*Koshihikari*’, and ‘*Zhenzhuhong*’ were obtained from Jilin Academy of Agricultural Sciences (Changchun, China). After sterilization by 1% (*v*/*v*) sodium-hypochlorite for five minutes, the seeds were rinsed by sufficient distilled water and immediately germinated for 48 h. Then, the germinated seeds were transferred to a 250 mL-baker with 100 mL ½Hoagland medium [24] and kept in 25 °C illumination incubator with a photoperiod of 16 h light and 8 h dark until they acquired relatively mature roots. 

### 2.3. Root Surfaces Characterization

#### 2.3.1. Scanning Electron Microscope (SEM)

The morphology of root surfaces was recorded using a JSM-7800F (JEOL, Tokyo, Japan) scanning electron microscope (SEM). Before SEM measurements, rice root tissues (root hair zone) were gradually dehydrated by 30%, 50%, 70%, 90% and 95% ethanol for 20 min, and finally washed with 100% ethanol twice. Root surfaces were coated with a layer of gold, and the images were taken.

#### 2.3.2. Water Contact Angle

The hydrophobicity of root surfaces was determined by [25,26], as follows: the cultivated rice root was placed on sterile filter paper until dry, followed by fixation onto the slide with double-sided tape. The contact angles were measured by the sessile drop method using a video-based optical contact angle measuring device DSA25 (Kruss, Germany). DDI water droplets (1.0 μL, *n* = 6) were deposited on the fixed roots in distances of a few millimeter. The shape of each drop was captured in a video sequence of which the contact angle after 5 s was evaluated using the Advance software (Kruss ADVANCE 1.7.2.1, Germany). Glass surfaces and polystyrene surfaces were evaluated as control.

#### 2.3.3. Initial Adhesion Measurement

Dried roots of rice (cut into 1 cm length), glass and polystyrene slides were placed in 24-well plates with one sample per well. The bacterial JM2-gfp was cultured in Luria-Bertania (LB) by rotating oscillator at 30 °C and 180 rpm to mid-log-phase, and harvested by centrifugation at 8000 rpm for 5 min at 4 °C, followed by washing three times with PBS (pH 7.2). The final pellets were resuspended in PBS and adjusted to OD_600 nm_ = 0.1. All OD measurements were determined using a spectrophotometer Infinite200pro (Tecan, Switzerland). To each well was added 2 mL cell suspension and then incubated at 25 °C for 16 h. After incubation, non-adhered cells were removed by washing with sterilized PBS three times; the immobilized cells were fixed by 2.5% glutaraldehyde, and washed with PBS. Slices of peeled root surface, glass and polystyrene were imaged by CLSM (FLUOVIEW FV 1000, Olympus, Tokyo, Japan). The captured images of bacterial adhesion on the surface of each substrate were then analyzed by ImageJ (Java 1.8.0). Six pictures were randomly taken for each sample [27,28].

### 2.4. Biofilm Formation

*Pseudomonas* sp. JM2-gfp cells were cultured in LB medium to the mid-log-phase, and then collected by centrifugation. The cell pellet was suspended in ½Hoagland medium. Rice roots were immersed in a suspension of the bacteria (OD_600 nm_ = 0.8) and incubated at 25 °C for four days under static conditions. The roots were harvested daily, and prepared for confocal laser scanning microscopy (CLSM, Olympus FV1000). 488 nm laser line of the argon laser and a 535 nm-long pass filter were used for excitation and emission, respectively. In addition, the dynamics of bacterial growth was determined by counting the total number of bacterial colonies after serial dilution and plating as follows: First the rice roots were removed from the culture solution during the biofilm formation process, and gently washed 2–3 times with sterile water to remove the planktonic bacteria. After drying with absorbent paper the roots were weighed. Then, the rice roots were washed with sterile water in a clean bench, blotted with sterile filter paper, and placed in a sterile centrifuge tube. An appropriate amount of sterile water was added into the tube before vigorous vortex to completely destroy the biofilm on the rice root. A suspension containing free bacteria was subsequently obtained and subject to serial dilution of 10^−3^, 10^−4^ and 10^−5^ with sterile water. Finally, 100 μL of the diluted bacterial suspension was taken and uniformly coated on the LB-resistant plate. Counting was performed after incubation at 30 °C for 18 h. To confirm that the biofilm was completely removed, the root was examined under the microscope after the removal of residual liquid on its surfaces [5,29,30]. All the experiments were repeated three times.

### 2.5. Characterization of the Biofilm on Root Surfaces

#### 2.5.1. Scanning Electron Microscope (SEM)

The morphology of biofilms attached to surfaces was recorded using a JSM-7800F (JEOL, Tokyo, Japan) scanning electron microscope (SEM). Before SEM measurements, rice roots, glass and polystyrene substrates were immersed in a suspension of the bacteria (OD_600 nm_ = 0.8) and incubated at 25 °C for two days, respectively. Samples were cut into 5 mm × 10 mm pieces and covered with a layer of gold before imaging.

#### 2.5.2. Confocal Laser Scanning Microscopy (CLSM)

Images of the biofilms formed on the surface of rice root, glass and polystyrene were captured using a confocal laser scanning microscopy (CLSM) system (FLUOVIEW FV 1000, Olympus, Tokyo, Japan). A series of images were obtained at 1μm intervals in the z section for a three dimensional view of the biofilm. At least five representative optical fields were examined for each specimen.

About 10 image stacks were collected randomly from different points and analyzed using the COMSTAT software [31]. The architecture properties of biofilms analyzed by COMSTAT included total biomass (μm^3^·μm^−2^), average thickness (μm), roughness coefficient, average diffusion distance (μm), and surface to biovolume ratio (μm^2^·μm^−3^). All the experiments were performed in triplicate.

#### 2.5.3. Composite Extracellular Polymeric Substances (EPS) Characterization

Biofilm associated EPS was extracted based on the method of Mangwani et al [32]. The specific steps are as follows. First, JM2-gfp cultured to mid-log-phase in LB medium was centrifuged and adjusted to OD_600 nm_ of 0.8 with 1/2 hoagland medium. Next, 10 mL of cultures was transferred to flasks with different materials. While in control group, 10 mL of 1/2 hoagland medium, instead of the culture, was added into the flask. Then, after incubation for 48 h at 30 °C, planktonic cells were carefully aspirated from the flasks. The spent medium and unattached cells were also removed from the substrate by rinsing with sterile PBS 2–3 times. Then, 10 mL of PBS was added to the flasks before gentle vortex to disintegrate the biofilm from the substrate. Afterwards, the sample was collected and centrifuged at 6500 g and 4 °C for 20 min. Then, the supernatant was collected, mixed with double volume 90% frozen ethanol, and kept at 4 °C for 18 h. Finally, the supernatant was discarded by centrifugation at 10,050 g for 10 min at 4 °C, and the precipitate was dried at 60 °C to remove ethanol. The composition of the biofilm EPS was analyzed using fourier transform infrared spectroscopy (FTIR) (Nicolet-iS5, Thermo). Total carbohydrate and protein content of the extracted EPS was estimated by the phenylsulphuric acid method and Bradford method, respectively. EPS on the surface of 12 rice roots, 10 pieces of glass or polystyrene after biofilm formation were evaluated for protein and carbohydrate content [33].

### 2.6. Degradation of Phenanthrene by the Biofilm

The roots were incubated for the formation of biofilms according to 2.4. The plants with bacterial biofilm on root (12 plants per flask) (CWB) were incubated in the 100 mL of 1/2 Hoagland medium with phenanthrene (50 mg/L) at 30 °C under static conditions (dark) for a period of time. Plants without bacteria (CW) were introduced as controls. The initial volume of the medium was maintained for each treatment throughout the incubation by adding fresh Hoagland medium to the culture flasks each day. After 10 days, phenanthrene was extracted from the plant tissues as previously described [34]. In brief, the concentration of phenanthrene was measured by HPLC (Agilent, 1200) equipped with a reverse-phase C18 column (Agilent, 5 μm, 250 × 4.6 mm) with methanol and water (*v*/*v* = 90:10) as mobile phase at a flow rate of 1 mL·min^−1^. Chromatography was performed at 25 °C. The phenanthrene was detected at 245 nm. The injection volume was 10 μL. 

The phenanthrene residue A in rice tissues was calculated by the following formula: A = Ci × M, where Ci represents the concentration of phenanthrene (mg/kg) in rice roots or stems and leaves, M represents the dry weight of rice roots or stems and leaves (g/bottle) 

### 2.7. Statistics

One-way variance analysis (ANOVA) was used for statistical analysis of means and standard deviations using Duncan’s Multiple Range Test (*p* < 0.05) to detect significant difference between mean values. All data were presented as mean ± SD.

## 3. Results and Discussion

### 3.1. Morphology of Rice Root Surfaces

**Figure 1:**
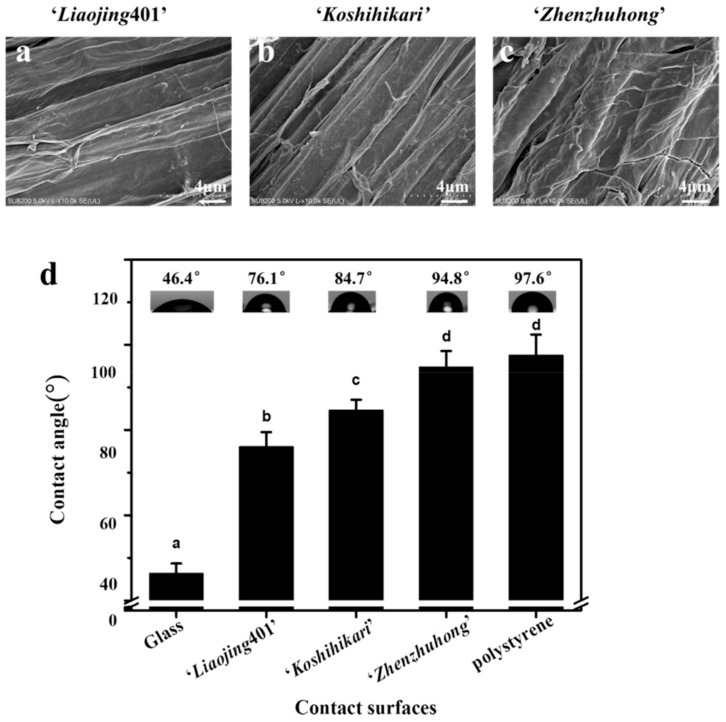
Morphology of the rice root surfaces (‘*Liaojing*401’, ‘*Koshihikari*’, ‘*Zhenzhuhong*’) using scanning electron microscopy (SEM) (**a**–**c**) and the hydrophobicity of contact surfaces (glass, ‘*Liaojing*401’, ‘*Koshihikari*’, ‘*Zhenzhuhong*’ and polystyrene) determined using a contact angle meter (**d**). The lowercase letters on top of the bars indicate significant differences in the means of contact angle (*P* < 0.05). Error bars are shown as ± SD.

Morphologies of root surfaces from three species of rice were observed by scanning electron microscopy (SEM) (Figure 1a–c). All the long sides of the root surfaces were parallel to the longitudinal axis of the roots with tight arrangement. The root surfaces presented wrinkle-like structures, providing large surface area to entrap bacteria. The structures may also facilitate the formation of biofilm by the phenanthrene-degrading bacteria JM2-gfp as they provide more anchoring points for the adhesion of bacteria.

### 3.2. Hydrophobicity Characterization

The hydrophobicity of root surfaces was evaluated by measuring the water contact angle with hydrophilic glass and hydrophobic polystyrene (abiotic surfaces) as controls. Among the rice species of ‘*Liaojing*401’, ‘*Koshihikari*’ and ‘*Zhenzhuhong*’, the root surfaces of ‘*Zhenzhuhong*’ presented the largest contact angle 94.8°, which was close to that of polystyrene surface (97.6°). Also, the root surfaces of ‘*Liaojing*401’ and ‘*Koshihikari*’ showed smaller contact angles, 76.1° and 84.7°, respectively (Figure 1d). In conclusion, the root surfaces of all the three rice species were hydrophobic. Similarly, Bonebrake et al [26] reported that the contact angle of wheat root surfaces after seven days of culture was 60.5°.

### 3.3. Initial Adhesion of Rice Root Surface

**Figure 2:**
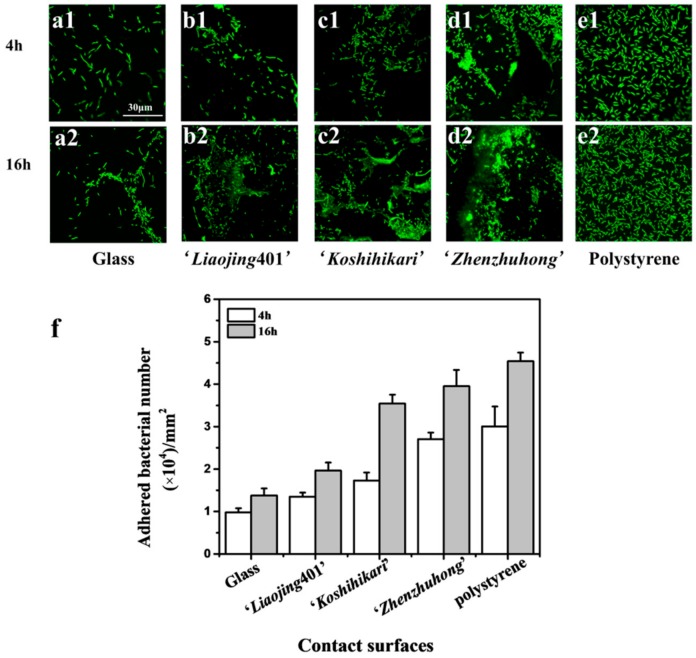
Laser confocal microscopy (CLSM) observation of the initial adhesion of JM2-gfp on the surfaces of glass, ‘*Liaojing*401’, ‘*Koshihikari*’, ‘*Zhenzhuhong*’ and polystyrene at 4 h (**a1**,**b1**,**c1**,**d1**,**e1**) and 16 h (**a2**,**b2**,**c2**,**d2**,**e2**). Quantitative analysis of JM2-gfp adhesion on different contact surfaces according to the fluorescence images (**f**). Error bars are shown as ± SD.

The initial adhesion of phenanthrene-degrading bacteria JM2-gfp on rice root surfaces was measured by CLSM as the first stage of biofilm formation. Hydrophilic surfaces of the glass showed the lowest initial adhesion at 4 h and 16 h (Figure 2(a1,a2)), while the hydrophobic surfaces of polystyrene showed the highest number of attached bacteria (Figure 2(b1,b2)). The adhesion of bacteria on rice root surfaces of ‘*Liaojing*401’, ‘*Koshihikari*’, ‘*Zhenzhuhong*’ increased with the hydrophobicity of substrate (Figure 2(c1,c2,d1,d2,e1,e2)). Moreover, the adhesion of JM2-gfp at 16 h was more significant than that at 4 h (Figure 2f). The biofilm formation is a stepwise and dynamical process consisting of initial attachment, irreversible attachment, early development of biofilm architecture, maturation, and dispersion [35]. The adhesion of bacteria to solid surfaces is a key step in the formation of biofilm [36]. It has been reported that hydrophobic interactions between the bacteria and the surfaces play a vital role in the initial adhesion. The hydrophobicity of the contacting surfaces affects bacterial attachment to the surfaces and the subsequent formation of biofilm [37]. One possible explanation for this is that on the hydrophobic substrate the interface water is removed faster that on the hydrophilic substrate when making initial contact with the bacteria [38]. The removal of interface water enables closer and attractive acid-base interactions [39]. It is reported that the hydrophobicity of zirconia affected the initial adhesion force and early attachment of *Streptococcus mutans* [40]. Moreover, the hydrophobic surfaces lead to an increased accumulation of cell-adhesion proteins at the interface, which act as specific binding sites for bacteria in order to accelerate and enhance bacterial adhesion [41,42].

### 3.4. Biofilm Formation on the Root Surfaces

**Figure 3:**
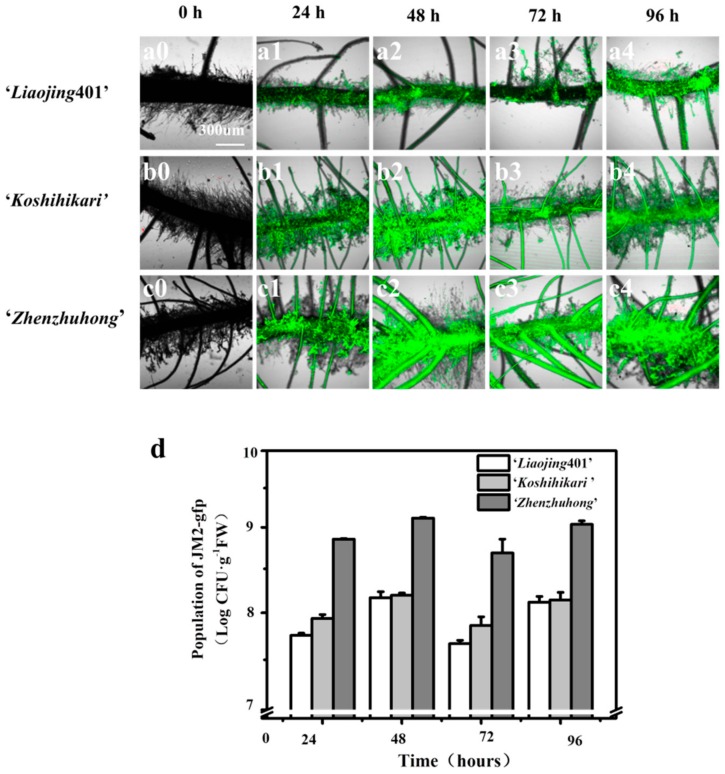
Biofilm formation by JM2-gfp on the root surfaces of ‘*Liaojing*401’ (**a0**,**a1**,**a2**,**a3**,**a4**), ‘*Koshihikari*’ (**b0**,**b1**,**b2**,**b3**,**b4**), ‘*Zhenzhuhong*’ (**c0**,**c1**,**c2**,**c3**,**c4**) within 96h observed using CLSM and plate counting (**d**). The images shown were produced from overlays of fluorescence (false-colored green) and transmitted light (gray) images.

The biofilm formation of strains JM2-gfp on the rice roots surfaces is shown in Figure 3. The CLSM images showed that JM2-gfp formed biofilm on the root surfaces of all three rice cultivars rapidly. With the adhesion of the bacterial cells on the surfaces of the roots, non-colonized spaces were gradually filled with bacteria or microcolonies. Ultimately, when the bacterial community started to grow in a three-dimensional manner, the biofilm became mature at 48 h (Figure 3a–c). The number of bacteria on the root surfaces of ‘*Liaojing*401’, ‘*Koshihikari*’ and ‘*zhenzhuhong*’ at 48 h were 8.17 logCFU·g^−1^, 8.20 logCFU·g^−1^ and 9.12 log CFU·g^−1^, respectively (Figure 3d). In addition, the density of the bacteria on the root surfaces slightly fluctuated due to the variation in the generation periods of biofilm (data not shown). 

### 3.5. Characterization of Biofilm on the Root Surfaces

#### 3.5.1. Morphology of biofilms

**Figure 4:**
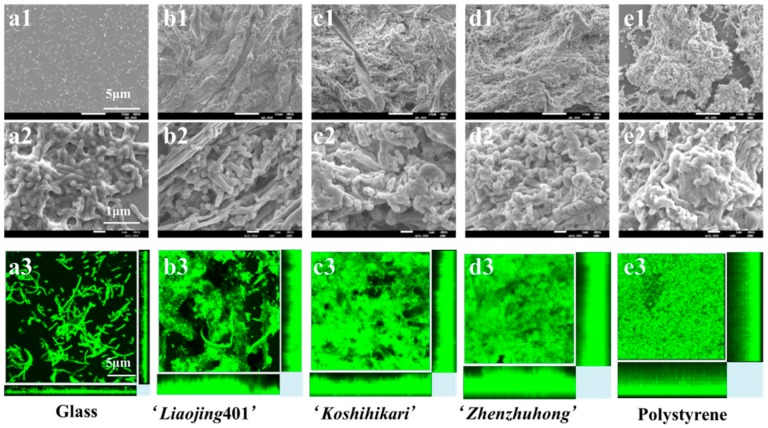
Morphological characterization of biofilm on the different surfaces of Glass, ‘*Liaojing*401’, ‘*Koshihikari*’, ‘*Zhenzhuhong*’ and polystyrene by SEM (**a1**,**b1**,**c1**,**d1**,**e1**; **a2**,**b2**,**c2**,**d2**,**e2**; 2 are enlarged views of 1) and by CLSM (**a3**,**b3**,**c3**,**d3**,**e3**).

Morphology of the biofilm formed by the strain JM2-gfp on different surfaces were imaged by SEM. Biofilms formed on glass, rice root surfaces (‘*Liaojing*401’, ‘*Koshihikari*’, ‘*Zhenzhuhong*’) and polystyrene exhibited different degrees of maturation at 48 h (Figure 4(a1–e1)). Higher magnification (10000×) images clearly showed that the biofilm on the hydrophilic glass was composed of numerous cells attached to the surfaces, lacking dense EPS or compressed aggregation (Figure 4(a2–e2)). However, biofilm presented on the hydrophobic polystyrene had dense cell aggregates and biomass embedded in EPS matrix. Similar to that on the polystyrene, robust biofilms were formed on the rice roots of ‘*Liaojing*401’, ‘*Koshihikari*’ and ‘*Zhenzhuhong*’, possibly due to their hydrophobicity. On the root surfaces of ‘*Zhenzhuhong*’ the biofilm showed the highest density of tightly intertwined bacteria among the three rice species in particular (Figure 4(a3–e3), Table 1). Moreover, biofilm formed on the plant root surfaces exhibited more extracellular matrix than that on the polystyrene surfaces (Figure 4(a1–e1)), probably because root tissues are constantly exuding chemicals such as various carbohydrates, amino acids, organic acids, as well as other absorbed compounds essential for the growth of root [43,44].

#### 3.5.2. Characterization of Bacterial Biofilm

Compared with conventional quantification methods, COMSTAT analysis carries the advantage of analyzing the biofilm without disturbing its structure [31]. The volumetric parameters of microbial biofilm architecture on the root surfaces including total biomass, roughness coefficient, surface to biovolume ratio, average diffusion distance, and average thickness were characterized using the COMSTAT program (Figure 4(a3–e3) and Table 1). 

Total biomass represents the overall volume of the biofilm, and it can be defined as the biomass volume divided by substratum area. The strain JM2-gfp showed the maximum total biomass on the polystyrene surfaces (7.519 ± 0.548 μm^3^·μm^−2^), declined in turn were the root surfaces of ‘*Zhenzhuhong*’, ‘*Koshihikari*’, ‘*Liaojing*401’ (6.387 ± 0.470, 5.121 ± 0.132, 4.400 ± 0.331 μm^3^·μm^−2^). Glass surfaces showed low biomass accumulation (0.022 ± 0.005 μm^3^·μm^−2^).

The roughness coefficient (R*) provides a measure of how structured the biofilm architecture is. The value of R* of the biofilm on glass was above 1, indicating biofilm heterogeneity and bacterial growth as microcolonies [45]. However, the R* of the biofilms on the roots of ‘*Liaojing*401’, ‘*Koshihikari*’, ‘*Zhenzhuhong*’ and polystyrene were less than 0.1, indicating a less heterogeneous architecture.

Surface to biovolume ratio also indicates portion of the biofilm, Glass (0.113 ± 0.014 μm^2^·μm^−3^) had lower ratios than the roots of ‘*Liaojing*401’, ‘*Koshihikari*’, ‘*Zhenzhuhong*’ (1.125 ± 0.195, 1.297 ± 0.088, 1.495 ± 0.521 μm^2^·μm^−3^) and polystyrene (1.500 ± 0.333 μm^2^·μm^−3^), suggesting differential capabilities to access the limited supply of nutrients [46]. 

The diffusion distance is the average of the distances among all biomass pixels, providing a measure of the distances over which nutrients and other substrate components must diffuse from the voids to the microorganism within microcolonies [46]. The polystyrene substrate and ‘*Zhenzhuhong*’, ‘*Koshihikari*’, ‘*Liaojing*401’ root surfaces had higher average diffusion distances (1.265 μm, 1.034 μm, 1.006 μm and 0.923 μm) than that of glass (0.001 μm).

Average thickness provides information on the upper extent of the biofilm. Overall, the glass surfaces showed thinnest biofilms (12.7 ± 1.71 μm) than the hydrophobic materials polystyrene (31.7 ± 2.11 μm), ‘*Liaojing*401’ (21.5 ± 0.95 μm), ‘*Koshihikari*’ (25.8 ± 2.45 μm) and ‘*Zhenzhuhong*’ (30.9 ± 2.54 μm). Moreover, the average thickness of the biofilm and the coverage of bacteria increased with the increase of hydrophobicity in 48 h. Taken together, the biofilm development on the root surfaces investigated in this study demonstrated that the characteristics of the substrate surfaces influenced the biofilm structure, which was in accordance with previous studies. It was observed that *Salmonella* strains adhered more easily to hydrophobic materials (such as polystyrene) than to glass [11,13]. In addition to *Salmonella*, *Pseudomonas aeruginosa* and *Pseudomonas putida* also showed preference to hydrophobic substrates during biofilm formation. It was reported that on titanium (Ti), a metal substrate with lower hydrophobicity than zirconia (ZrO_2_), both the biomass and the density of *Pseudomonas aeruginosa* biofilm were lower than those on ZrO_2_ [47]. Similarly, the hydrophobicity of polymer increased *Pseudomonas putida* adhesion, as well as the dynamics and structure of formed biofilm [48]. Bacterial adhesion of isolated *Pseudomonas* was enhanced when attached onto RO membranes with higher hydrophobicity [49,50]. However, it should be pointed that many other factors also influence the formation of biofilm, including strains, culture media and temperature of incubation [15]. Moreover, these factors along with the method used for biofilm quantification can cause the variations observed among different reports [37,51].

#### 3.5.3. Characterization of Biofilm Associated EPS

**Figure 5:**
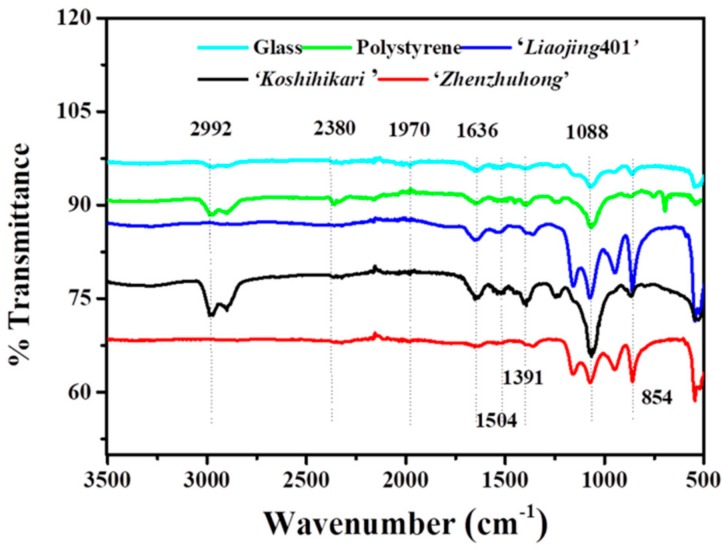
FTIR characterization of the biofilm extracellular matrix on glass, ‘*Liaojing*401’, ‘*Koshihikari*’, ‘*Zhenzhuhong*’ and polystyrene.

Biofilm associated EPS provides structural and functional benefits to the biofilm, such as hydration, resource capture, digestive capacity and protection from the environment, in addition to facilitating intercellular interactions that enhance the metabolic capacity of cells in the biofilm [52]. The composition of the EPS matrix within bacterial biofilms has been demonstrated to be highly variable, depending upon the environment and the substrate upon which the EPS are formed [53]. The composition of functional groups on EPS was analyzed by FTIR spectroscopy (Figure 5). The peaks represent the functional groups -COC-, PO^4−^, -NH_2_ and -CH_2−_, indicating the presence of polysaccharides, nucleic acids, proteins and lipids in the EPS. The band in the range of 1320–1000 cm^−1^ indicated the existence of uronic acid in EPS. The peak between 3200 and 2800 cm^−1^ indicated the presence of lipids in EPS. The existence of carbohydrates was confirmed by the -COC- stretch between 1750 and 1625 cm^−1^. Characteristic peaks of the amide were observed at 1650–1600 cm^−1^, which was assigned to the stretching vibration of N–H demonstrated the presence of protein. The peak at 1238 cm^−1^ indicated the phosphate groups of nucleic acid [54]. Taken together, the FTIR results suggested that proteins, carbohydrates, lipids, nucleic acids were present in the EPS of biofilms formed on different substrates including rice root surfaces.

Carbohydrates and proteins are the basic structural elements of EPS, accounting for more than 95% weight, which determine the mechanical stability of biofilm, mediate and consolidate cell adhesion, as well as promote cell-cell aggregation [55]. The content of protein and carbohydrate in biofilms formed on different hydrophobic surfaces was quantitatively analyzed by Bradford method and phenol sulfuric acid method. Since the surface area of rice root is not well quantified, the bioactive interface (rice root surfaces) and non- bioactive interfaces (glass and polystyrene) fit different quantitative units (Table 2). The total carbohydrate and protein content of EPS on the hydrophobic polystyrene were higher than that on the hydrophilic glass. In addition, extracellular proteins and carbohydrates were positively correlated with the hydrophobicity of root surfaces, with the highest amounts 531.9 μg and 309.8 μg presented on the root surfaces of ‘*Zhenzhuhong*’, and the lowest 323.2 μg and 258.4 μg on root surfaces of ‘*Liaojing*401’, respectively. Our results indicated that the hydrophobicity of the root surfaces affected the content of proteins and carbohydrates in the extracellular matrix secreted from phenanthrene-degrading bacteria, with underlying mechanisms left to be further explored. In conclusion, for practical application, it is crucial to choose and/or modify a highly hydrophobic crop root to increase the excretion of EPS, and to promote the structural stability of the biofilm. Additionally, it was reported that the EPS contents in biofilms showed significant correlations with the biodegradation of PAHs [56]. 

### 3.6. Capacity of the Phenanthrene Degradation by the Biofilm on Rice Root Surfaces

**Figure 6:**
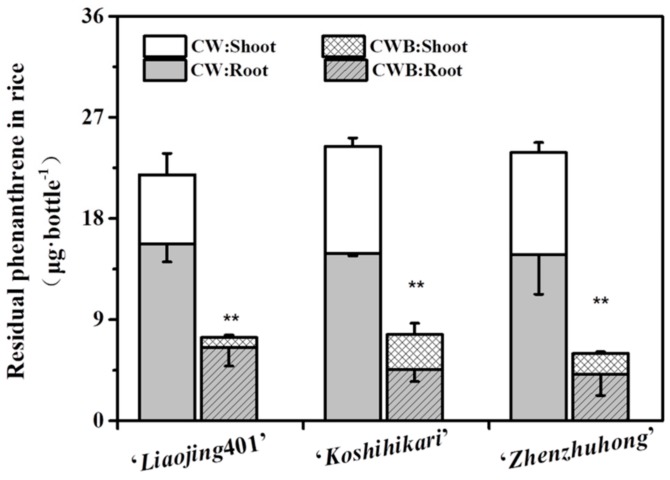
The residual phenanthrene in ‘*Zhengzhuhong*’, ‘*Koshihikari*’ and ‘*Liaojing*401’ root and shoot. plants cultured in phenanthrene-containing Hoagland medium (CW); plants with biofilm cultured in phenanthrene-containing Hoagland medium (CWB). Error bars are shown as ±SD.

The phenanthrene-degrading function of biofilm was studied in a hydroponic system. Rice plants with mature root-biofilm were exposed to 1/2 Hoagland medium with initial concentration of phenanthrene 50 mg·L^−1^. After 10 days of incubation, more phenanthrene was degraded by the biofilm-colonized plants compared with the control plants (Figure 6). The residual concentration of phenanthrene in biofilms formed on the roots of ‘*Zhengzhuhong*’, ‘*Koshihikari*’ and ‘*Liaojing*401’ were significantly lower than those in the corresponding CW groups, showing a 71.9%, 69.3% and 58.7% decrease, respectively. Correspondingly, the residual concentrations of phenanthrene in the shoots were decreased by 85.5%, 79.9% and 67.5%. Compared with the previously reported degradation results of phenanthrene-degrading colonization roots, our bacteria biofilms on root exhibited higher degradation efficiency. The accumulations of phenanthrene in *Pseudomonas* sp. Ph6-gfp-colonized ryegrass via soaking, root soaking and leaf painting were 20.1%, 33.1% and 7.1%, respectively [34]. The concentrations of phenanthrene in *Sphingobium* sp. RS2-gfp-inoculated plants was reduced by 39.9% and 62.5% in roots and shoots via bud soaking and by 25.1% and 35.8% via root inoculation as compared to the original concentration, respectively [5]. Mangwani et.al reported that extracellular polymeric substances govern the development of biofilm and mass transfer of polycyclic aromatic hydrocarbons for improved biodegradation [33,56,57]. Our results indicated that the root surfaces promote the structural stability of the bacterial biofilm production. 

Taking both our findings and the previous reports into consideration, we speculate that the content of EPS enhanced the solubility of hydrophobic organic compounds. Thus, the biofilm colonized on roots plays an important role in the degradation of phenanthrene, thereby reducing the phenanthrene uptake of plants and further lowering the risk of plants contamination.

## 4. Conclusions

Our study aims to develop a biofilm by phenanthrene-degrading bacteria on the plant root for the establishment of a system controlling organic pollutant transportation into the plant. The hydrophobicity of the root surfaces of three rice species ‘*Liaojing*401’, ‘*Koshihikari*’, and ‘*Zhenzhuhong*’ increased the initial adhesion of strain JM2-gfp. The robust and crowded homogeneous volumetric biofilm associated with the EPS matrix was obtained on the rice root surfaces at 48 h. The proteins, carbohydrates, lipids, nucleic acids were produced in the EPS. Taken together, the biofilm colonized on roots plays a vital role in the degradation of phenanthrene and prevention of phenanthrene uptake in plants. Moreover, drastic reductions were also observed in the shoots. In summary, our work provides valuable insights into the novel application of biofilm for the management of environmental risks caused by PAHs in the crop products in contaminated agriculture soils. 

## Figures and Tables

**Table 1:** Confocal laser scanning microscopy parameters of JM2-gfp biofilm on the surfaces of glass, ‘*Liaojing*401’, ‘*Koshihikari*’,‘*Zhenzhuhong*’and polystyrene after 48 h of growth. Data are shown as mean ± SD.

Biofilm Parameters	Glass	‘*Liaojing*401’	‘*Koshihikari*’	‘*Zhenzhuhong*’	Polystyrene
Total biomass (μm^3^·μm^−2^)	0.022 ± 0.005	4.400 ± 0.331	5.121 ± 0.132	6.387 ± 0.470	7.519 ± 0.548
Roughness coefficient	1.975 ± 0.005	0.057 ± 0.007	0.059 ± 0.013	0.056 ± 0.011	0.005 ± 0.001
Surface to biovolume Ratio (μm^2^·μm^−3^)	0.113 ± 0.014	1.125 ± 0.195	1.297 ± 0.088	1.495 ± 0.521	1.500 ± 0.333
Average diffusion distance (μm)	0.001 ± 0.000	0.923 ± 0.041	1.006 ± 0.119	1.034 ± 0.000	1.265 ± 0.197
Average thickness (μm))	12.70 ± 1.71	21.50 ± 0.95	25.80 ± 2.45	30.90 ± 2.54	31.70 ± 2.11

**Table 2:** The protein content and carbohydrate content in the extracellular matrix of biofilm formed on different surfaces were determined by Bradford method and phenol sulfuric acid method. Data are shown as mean ± SD.

	Protein Content (μg)	Carbohydrates Content (μg)
Glass (10 cm^2^)	366.0 ± 4.71	122.0 ± 1.19
Polystyrene (10 cm^2^)	555.6 ± 3.71	149.1 ± 0.92
‘*Liaojing401*’ (12 per)	323.2 ± 3.94	258.4 ± 0.79
‘*Koshihikari*’ (12 per)	426.4 ± 5.17	272.2 ± 1.28
‘*Zhenzhuhong’* (12 per)	531.9 ± 6.50	309.8 ± 0.97
